# Impact of time to local recurrence on the occurrence of metastasis in breast cancer patients treated with neoadjuvant chemotherapy: A random forest survival approach

**DOI:** 10.1371/journal.pone.0208807

**Published:** 2019-01-23

**Authors:** Enora Laas, Anne-Sophie Hamy, Anne-Sophie Michel, Nabilah Panchbhaya, Matthieu Faron, Thanh Lam, Sophie Carrez, Jean-Yves Pierga, Roman Rouzier, Florence Lerebours, Jean-Guillaume Feron, Fabien Reyal

**Affiliations:** 1 Department of Surgery, Institut Curie, Paris, France; 2 Residual Tumour & Response to Treatment Laboratory, RT2Lab, PSL Research University, Translational Research Department, Institut Curie, Paris, France; 3 INSERM, U932 Immunity and Cancer, Paris, France; 4 Department of Surgery, Institut Gustave Roussy, Villejuif, France; 5 Department of Gynaecology and Obstetrics, Geneva University Hospitals, Geneva, Switzerland; 6 Department of Medical Oncology, Institut Curie, Paris, France; 7 Paris Descartes University, Paris, France; University of South Alabama Mitchell Cancer Institute, UNITED STATES

## Abstract

**Background:**

We studied the relationship between time to ipsilateral breast tumor recurrence (IBTR) and distant metastasis-free survival (DMFS) in patients with breast cancer treated by neoadjuvant chemotherapy (NAC).

**Methods:**

Between 2002 and 2012, 1199 patients with primary breast cancer were treated with NAC. Clinical, radiological and pathological data were retrieved from medical records. Multivariate analysis was performed with the random survival forest (RSF) method, to evaluate the relationship between time to local recurrence and DMFS.

**Results:**

Time to IBTR, local recurrence and molecular subtype were the factors most strongly associated with DMFS. In the total population, DMFS increased linearly with recurrence time, up to 50 months. For recurrences after 50 months, DMFS was similar for all times to recurrence. Considering molecular subtypes separately, the threshold was similar for the TNBC subtype (50 months), but appeared to occur later for the luminal and *HER2*-positive subtypes (75 months).

**Conclusion:**

A threshold of 50 months seems to differentiate between early and late recurrences and could be used to guide the medical management of local breast tumour recurrences.

## Introduction

Neoadjuvant chemotherapy (NAC) is currently indicated as a means of allowing breast-conserving surgery in cases of breast carcinoma with a poor prognosis [1,2]. Studies comparing chemotherapy in adjuvant and neoadjuvant settings have reported similar prognoses for overall survival. However, consistent with the higher rates of conservative surgery in patients receiving NAC, local relapses are more frequent for NAC than for adjuvant chemotherapy, reaching 22% at 10 years [3–6].

Ipsilateral breast tumour recurrence (IBTR) is an independent risk factor for both distant metastasis [7–9] and death from breast cancer [10–12]. It is unclear whether this association is causal, whether IBTR is an indicator of active disease, or both. One of the major challenges in the management of local recurrences is distinguishing true recurrences, corresponding to the regrowth of resistant cells after initial treatment, from new primary tumours. This distinction is important for treatment, because true recurrences provide evidence of uncontrolled disease requiring radical treatment, rather than the secondary conservative surgery that could be considered for a new tumour. The risk of metastasis is also probably different for these two entities and should be considered as such for decisions concerning systemic treatment.

Time-to-recurrence seems to be a relevant surrogate for distinguishing “late recurrences” potentially corresponding to new primary tumours, from “early recurrences” more likely to correspond to progression of the initial disease. Some studies of adjuvant chemotherapy have reported better outcomes for patients with “late recurrences” than for those with “early recurrences”[13–17]. However, only two studies have investigated the impact of time to local recurrence on distant metastasis-free survival in the neoadjuvant setting [9,18]. One limitation common to all these studies is that the threshold for distinguishing between early and late recurrences is chosen by the author and often defined arbitrarily as the median time to recurrence, a time point that does not necessarily separate subgroups with good and poor prognoses. The accurate classification of IBTRs as “early” or “late”recurrences is essential in the neoadjuvant setting, to improve both prognostic evaluations and therapeutic choices.

Traditional statistical techniques, such as Cox’s proportional hazards (PH) models, are generally used to identify potential risk factors. However, Cox models involve restrictive assumptions, such as a proportionality of hazards and linearity [19]. These assumptions may bias the analysis of prognosis in the long-term follow-up of breast cancer and hinder the identification of early or late markers of prognosis [20,21]. For this reason, Baulies *et al*. introduced a time-dependent effect into their analysis, and showed that early local recurrence, within five years, in patients treated by conservative surgery, was a prognostic factor strongly associated with the development of distant metastases [18].

However, the relationships between clinical outcome and the predictors considered are potentially complex. It may be difficult to identify interactions, particularly those involving multiple variables, such as three-way interactions. This complexity would bias the relationship between IBTR, the time to local recurrence (time to IBTR) and distant metastasis-free survival (DMFS). These difficulties can be handled automatically by machine-learning methods, such as tree-based approaches. Random survival forests (RSFs) are a non-parametric tree-based ensemble learning method that can be used to select and rank variables [19,22–25] without the limitations of Cox models. One of the key advantages of the RSF approach is the adaptive discovery of nonlinear effects and interactions. This approach uses all the available variables in the dataset to build the response predictor, without the need for explicit specification of the functional form of the covariates. Several studies have confirmed that RSF methods perform better in this context than the traditional Cox PH model [22,26,27].

We used the RSF method to study the relationship between time to IBTR, distant metastasis-free survival (DMFS) and overall survival (OS) survival in a large series of breast cancer patients treated with NAC.

## Materials and methods

NEOREP (“Reponse à la chimiothérapie neoadjuvante”)(Cohort, CNIL declaration number 1547270) is a retrospective cohort follow-up study of patients treated with NAC for a unifocal invasive breast carcinoma at Institut Curie (Paris, France) between 2002 and 2012. All experiments were performed retrospectively and in accordance with the French Bioethics Law 2004–800, the French National Institute of Cancer (INCa) Ethics Charter and after approval by the Institut Curie review board and ethics committee (Comité de Pilotage of the Groupe Sein). In the French legal context, our institutional review board waived the need for written informed consent from the participants. Moreover, women were informed of the research use of their tissues and did not declare any opposition for such researches. Data were analyzed anonymously. All cases were eligible for inclusion.

Clinical, radiological and pathological data, such as patient age, menopausal status, T stage, N stage, histological tumour grade, oestrogen receptor (ER), progesterone receptor (PR) and HER2 status, and pathological response to NAC, were recorded.

The pathological diagnosis was confirmed in all patients by a core needle biopsy before treatment. Histological grade was determined as described by Elston and Ellis (1991), with a modified version of the Scarff–Bloom–Richardson grading system. Hormone receptors were analysed by immunohistochemistry. Tumours were considered to be positive for ER or PR if 10% of the carcinomatous cells displayed positive staining, as recommended by European guidelines [28]. *HER2* expression status was determined according to American Society of Clinical Oncology guidelines [29]. The molecular subtype of each tumour was determined as follows: “luminal” for breast tumours positive for ER and/or PR, with no overexpression of *HER2*; “*HER2*-positive” for tumours with *HER2* overexpression; and “triple-negative” for tumours displaying no staining for ER or PR and without *HER2* overexpression.

Patients were treated in accordance with national guidelines. Neoadjuvant chemotherapy regimens changed over time (anthracycline-based or sequential anthracycline-taxane regimens).

Surgery was performed four to six weeks after the end of chemotherapy. Patients underwent either mastectomy or breast-conserving surgery (lumpectomy), with axillary lymph node dissection (ALND) or sentinel node dissection (SLNB). SNLB, when indicated, was performed after NAC. In case of positive node in FNA, we performed ALND. A pathologic complete response (pCR) was defined as the absence of residual invasive cancer cells in the breast and axillary lymph nodes (ypT0is/ypN0).

After surgery, adjuvant treatment was administered in accordance with Institut Curie Treatment Guidelines. Adjuvant chemotherapy was administered at physician choice, according to the pathological response to NAC and lymph node status. The adjuvant chemotherapy chosen was with 5FU-Navelbine (FUN) in most of the cases. Trastuzumab was administered to patients with overexpression of HER2 since 2005. Patients received adjuvant radiotherapy according to national guidelines. Radiation was given in case of lumpectomy or in case of radical mastectomy for patient with initial T3 or T4 tumor, for all patients with involved axillary lymph nodes and to a selection of high-risk node-negative breast cancer patients.

Endocrine therapy (tamoxifen, aromatase inhibitor, or GnRH agonists) was added to the regimen as an adjuvant treatment, for almost all hormone receptor-positive tumours. After the completion of treatment, the patients were followed up every four months for the first two years, every six months for the next three years and then annually from the fifth year onwards. Clinical examination, mammography and breast ultrasound were performed annually.

Time to IBTR was defined as the time from diagnosis to local recurrence in the previously treated breast, and was measured from the date of diagnosis to the time of the last follow-up visit or IBTR. Distant metastasis-free survival (DMFS) was defined as the time from diagnosis to distant recurrence or to the last follow-up visit, whichever occurred first. Overall survival (OS) was defined as the time from diagnosis to death or to the last follow-up visit, whichever occurred first.

Patients for whom none of these events were recorded were censored at the date of last known contact.

### Statistical method

As the traditional Cox model was not the most suitable for our question, multivariate analysis was performed with the random survival forest (RSF) method, a nonparametric approach to survival analysis [19]. A set of survival trees of similar size was first built by recursive partitioning on a training set obtained from the original data set by random aggregation through bootstrap sampling (with replacement) of the data (bagging). Each tree was tested on the remaining group (the validation set). At each node of the tree, we randomly selected a subset of predictors as candidate variables for splitting, making the forest robust to correlations between predictors. In each tree, survival time and patient status were treated as response variables. Each RSF run was performed on 3000 trees, with the log-rank splitting rule and five predictors randomly selected at each split. Missing data were treated by a multiple imputation strategy. We analysed 16 clinical and histological variables: age, body mass index (BMI), menopausal status, clinical T and N stage, surgery type (lumpectomy or mastectomy), histological subtype, initial tumour size, initial tumour grade, initial Ki67 status, molecular subtype, margin status, pCR, postoperative nodal involvement (ypN stage), local recurrence and time to IBTR.

The importance of each of the model covariates was determined by internal variable ranking measures: variable importance (VIMP) and minimal depth. VIMP is the difference in validation set prediction error before and after the permutation of variables: the larger the VIMP, the more predictive the variable. A VIMP close to zero indicates that the variable makes little or no contribution to predictive accuracy, and negative values indicate that predictive accuracy is improved by omission of the variable.

Minimal depth indicates the impact of the variable on the prediction. The smaller the minimal depth, the more predictive the variable, as the variables with the smallest minimal depths split the largest samples of the population, frequently at nodes close to the root node.

VIMP and minimal depth may rank the variables differently. The variables selected had concordant values for VIMP and minimal depth, and a high predictive value.

Partial dependence plots were used to describe the adjusted predicted response to the covariate of interest, by integrating out the effects of variables other than the covariate of interest. Analyses were performed for the total population, and then for the three molecular subtypes separately.

Analyses were performed with R software version 3.2.2, with the R package "*random-ForestSRC*" (R Development Core Team, 2011,http://www.R-project.org/).

## Results

We included 1199 patients from the NEOREP cohort: 530 (44.2%) with luminal tumours, 375 (31.3%) with triple-negative tumours and 294 (24.5%) with *HER2*-positive tumours. Lumpectomy was performed in 66.6% (*n* = 797) of the patients. Pathological complete response (pCR) was achieved in 292 patients (24.4%) (Table 1). IBTR occurred in 89 patients (7.4% which 17 were regional recurrences) and distant metastasis occurred in 214 patients (17.8%). Twenty-three patients underwent SLNB following by ALND for positive node.

**Table 1:** Clinical, histological and follow-up characteristics of patients.

		All (n = 1199)	Luminal (n = 530)	Triple negative (n = 375)	*HER2*-positive (n = 294)	*p-value*
Age mean (sd)		48.5 (10.1)	48.7 (9.3)	48.4 (10.3)	48.4 (11.2)	0.84
N(%)	≥40 yrs	934 (78)	441 (83.2)	280 (74.7)	213 (72.9)	0.0005
	<40 yrs	263 (22)	89 (16.8)	95 (25.3)	79 (27.1)	
BMI mean (sd)		24.7 (4.7)	24.8 (4.8)	24.8 (4.6)	24.49 (4.5)	0.56
N(%)	<20	141 (11.8)	67 (12.7)	40 (10.7)	34 (11.6)	0.55
	20–30	895 (75)	383 (72.7)	288 (77)	224 (76.7)	
	>30	157 (13.2)	77 (14.6)	46 (12.3)	34 (11.6)	
Postmenopausal		442 (37.2)	184 (35)	143 (38.5)	115 (39.2)	0.4
Clinical size (*mm)*	≤20 mm	91 (7.6)	28 (5.3)	32 (8.5)	31 (10.5)	0.017
	>20 mm	1107 (92.4)	501 (94.7)	343 (91.5)	263 (89.5)	
Histology	Ductal	1062 (90)	448 (85.2)	339 (91.9)	275 (96.5)	<0.0001
	Lobular	74 (6.3)	65 (12.4)	6 (1.6)	3 (1.1)	
	Other	44 (3.7)	13 (2.5)	24 (6.5)	7 (2.5)	
EE-Grade	1	47 (4.1)	40 (7.8)	5 (1.4)	2 (0.7)	<0.0001
	2	432 (37.3)	287 (56.3)	48 (13.1)	97 (34.5)	
	3	678 (58.6)	183 (35.9)	313 (85.5)	182 (64.8)	
Mitotic Index	≤10	371 (34.3)	239 (50.6)	56 (16.3)	76 (28.6)	<0.0001
	11–22	331 (30.6)	136 (28.8)	94 (27.3)	101 (38)	
	>22	380 (35.1)	97 (20.6)	194 (56.4)	89 (33.5)	
	Missing*	117 (9.8)	58 (10.9)	31 (8.3)	28 (9.5)	
Clinical T stage	T1	70 (5.8)	21 (4)	31 (8.3)	18 (6.1)	0.11
	T2	798 (66.6)	361 (68.1)	242 (64.5)	195 (66.3)	
	T3	331 (27.6)	148 (27.9)	102 (27.2)	81 (27.6)	
Clinical N stage	N0	525 (43.8)	236 (44.6)	170 (45.3)	119 (40.5)	0.38
	N1	620 (51.8)	274 (51.8)	184 (49.1)	162 (55.1)	
	N2 or N3	53 (4.4)	19 (3.6)	21 (5.6)	13 (4.4)	
Breast surgery	Lumpectomy	797 (66.6)	306 (58)	289 (77.1)	202 (68.7)	<0.0001
	Mastectomy	400 (33.4)	222 (42)	86 (22.9)	92 (31.3)	
Axillary surgery	SLN dissection	27 (2.3)	1 (0.2)	18 (4.8)	8 (2.7)	<0.0001
	Axillary dissection	1114 (93.1)	509 (96.6)	339 (90.4)	266 (90.5)	
	Boths	55 (4.6)	17 (3.2)	18 (4.8)	20 (6.8)	
ypN+		657 (55.3)	359 (68.3)	143 (38.8)	155 (52.7)	<0.0001
Positive resection margin (lumpectomy)		31 (2.6)	16 (3.1)	8 (2.1)	7 (2.4)	<0.0001
Histologic size (mean, sd)		18.97 (18.29)	24.34 (18.2)	15.08 (17)	14.47 (17.7)	<0.0001
	*Missing*	112 (9)	56 (10.6)	25 (6.7)	31 (10.6)	
Pcr		292 (24.4)	35 (6.6)	142 (37.9)	115 (39.1)	<0.0001
IBTR		89 (7.4)	26 (4.9)	35 (9.3)	28 (9.5)	0.012
Time to IBTR	1 yr	22 (1.8)	3 (0.6)	19 (5.1)	0 (0)	<0.0001
	3 yrs	358 (29.9)	110 (20.8)	140 (37.3)	108 (37)	
	5 yrs	322 (26.9)	140 (26.4)	98 (26.1)	84 (28.8)	
	> 5 yrs	495 (41.4)	277 (52.3)	118 (31.5)	100 (34.2)	
Distant metastasis		214 (17.8)	100 (18.9)	80 (21.3)	34 (11.6)	0.003

*Only variables with >5% data missing are detailed

BMI: Body Mass Index; EE Elston-Ellis; IBTR Ipsilateral Breast tumour recurrence; pCR: Pathologic complete response; SLN: sentinel lymph node; yr: year; ypN+: positive post-operative nodes

The mean follow-up time was 59.8 months [56.5–62.1 months]. The median survival time was not reached for either lBTR time or DMFS.

**Fig 1:**
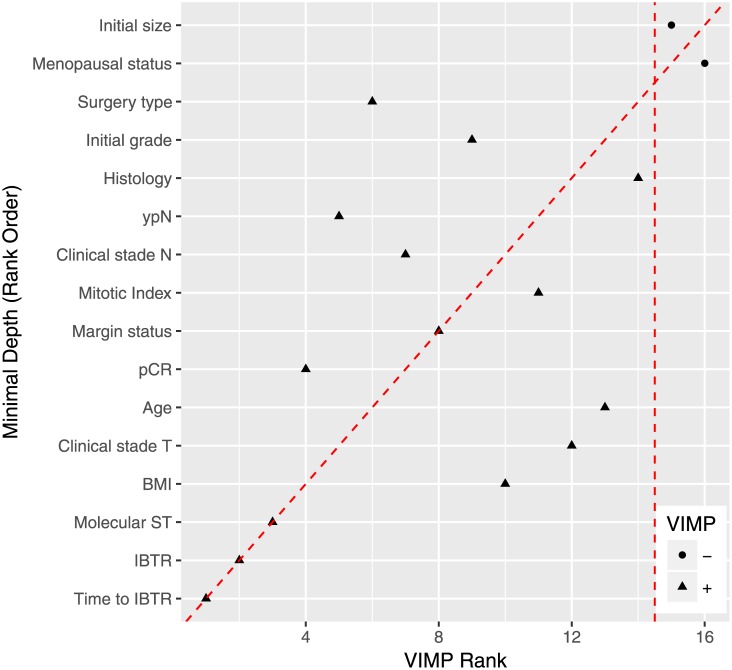
Minimal depth and VIMP rankings for covariate selection.

Fig 1 shows the VIMP and minimal depth of the variables obtained from the RSF for predicting DMFS. We tested 22 variables, of which time to IBTR, local recurrence and molecular subtype were the most predictive of DMFS, according to both VIMP and minimal depth values. Time to IBTR was the variable associated with DMFS with the lowest VIMP rank and minimal depth value.

**Fig 2:**
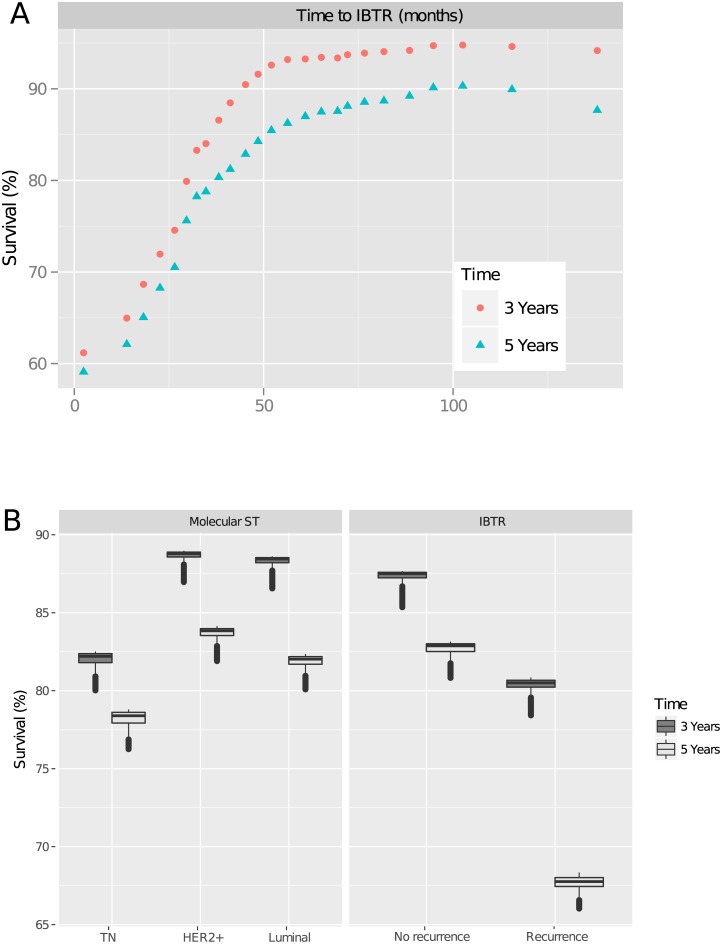
a. Time to ipsilateral breast tumour recurrence (IBTR). b. Ipsilateral breast tumour recurrence (IBTR) and molecular subtype.

Fig 2 shows the adjusted effect of the variables on DMFS. Three-year DMFS increased with time to recurrence (Fig 2A), reaching a plateau at a time to IBTR of 50 months. From this time to IBTR onwards, DMFS remained constant. A threshold of 50 months to IBTR was also appropriate for differentiating between early and late recurrence groups for five-year DMFS.

After adjustment for other variables, three- and five-year DMFS were lower in patients experiencing local recurrence, and in patients with TNBC, for longer times to recurrence (Fig 2B). Patients free from local recurrence had a three-year DMFS of 87% and a five-year DMFS of 83%, whereas patients experiencing recurrence had a three-year DMFS of 77% and a five-year DMFS of 64%. Three-year DMFS was similar for patients with luminal tumours and patients with *HER2*-positive tumours (about 88%), whereas TNBC patients had a three-year DMFS of 83%. At five years, DMFS was highest for patients from *HER2*-positive subgroup (84%), whereas TNBC patients had the worst prognosis (DMFS: 78%).

**Fig 3:**
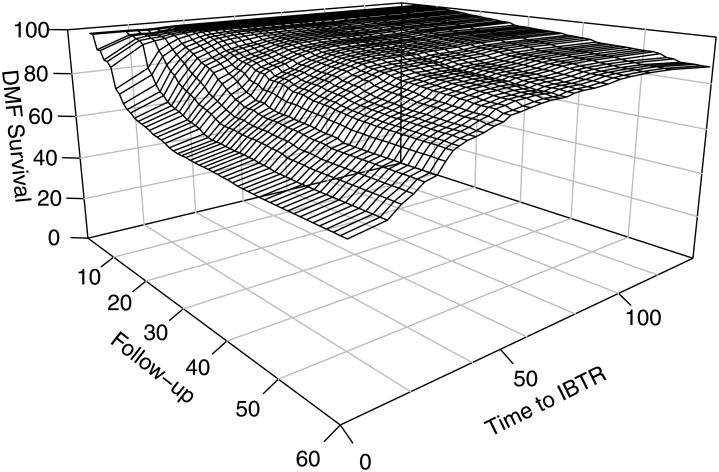
Partial dependence as a function of time estimated distant metastasis-free (DMF) survival at each follow-up time, for different times to ipsilateral breast tumour recurrence (IBTR).

Fig 3 shows the estimated DMFS at to the various time points in follow-up, for different times to IBTR.

This figure shows the effects of both follow-up time and time to IBRT on survival. The axis on the left shows how DMFS decreases over time, and that on the right shows how shorter times to IBTR affect survival. Patients with time to IBTR exceeding 50 months had a DMFS greater than 80% at 60 months of follow-up.

We defined a cut-off of 50 months for differentiating between early and late recurrences. For early recurrences (*i*.*e*. occurring between 0 and 50 months after NAC), a greater time to IBTR was associated with a higher DMFS, for all follow-up times considered.

DFMS was similar for all late recurrences (defined as occurring more than 50 months after the primary tumour), regardless of follow-up time, as shown by the plateau on the figure.

**Fig 4:**
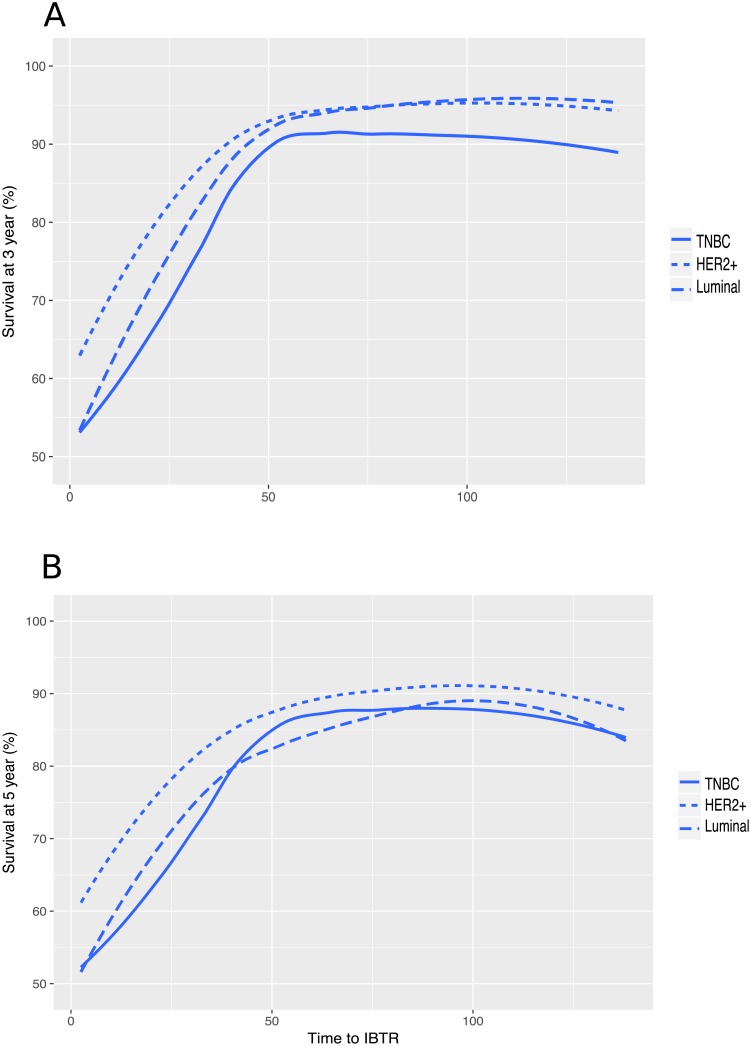
Partial dependence plots for time to IBTR, according to molecular subtype. a.at three years of follow-up. b.at five years of follow-up.

We then performed the same analysis separately for the three molecular subtypes (Fig 4A and 4B).

For all subtypes, three-year DMFS (Fig 4A) was lower for patients experiencing recurrence before 50 months than for those experiencing recurrence after 50 months. For five-year DMFS, the shape of the survival curve was similar to that for three-year DMFS for the TNBC subtype (cut-off at 50 months), whereas the cut-off point seemed to occur later for the luminal and the *HER2*-positive subtypes (after more than 75 months; Fig 4B).

## Discussion

Our results, obtained with a non-parametric approach, confirmed the prognostic importance of local recurrence, time to IBTR and molecular subtype for the occurrence of metastasis after NAC in breast cancer. Local recurrence and time to IBTR were the most important of the variables analysed for predicting patient outcome. Local recurrence has long been considered a poor prognosis factor *per se* [7–12]. Rouzier *et al*. found a relative risk of 5.34 (95% CI: 3.23–8.82) for the development of distant metastasis after local relapse in 257 patients treated by NAC and conservative surgery [9]. In this study, 59.7% of patients experiencing a local recurrence had developed distant metastases at five years.

Time to recurrence has been shown to be an important prognostic factor in early breast cancer [8,9,18,30–32], with patients with late recurrence having better outcomes than those with early recurrence [15]. However, the precise definition of “early” and “late” recurrence remains a matter of debate, with the threshold generally set at between two and five years [8,13,15,17,31,33]. An accurate definition of this threshold is essential, as it can be used to distinguish between true recurrences and new primary tumours, which should be managed differently. Chemotherapy is widely used for the treatment of recurrences. All international recommendations include the use of time to recurrence to guide treatment decisions. For local management practices, radical mastectomy could be reserved for cases of true recurrence (i.e. aggressive disease), whereas new primary tumours (with a non-aggressive profile) could be treated by secondary conservative surgery.

Baulies *et al*. showed, with a time-dependent variable, that local recurrence in patients treated by conservative surgery within the first five years was a prognostic factor strongly associated with the development of distant metastases (HR 4.21; 95% CI 2.89–6.11; *P*<0.001) [18]. Other studies using a three-year threshold to distinguish between early and late recurrences found that early recurrence was associated with a higher risk of distant metastases [9,15]. Fredrikson used a ROC curve to determine the best trade-off between specificity (66%) and sensitivity (62%) for local recurrence; they identified a cut-off point for the risk of death, at 2.3 years after surgery [33]. Gosset *et al*. identified 34 months as the cut-off for time to IBTR minimising the *p*-value for the relationship between time to IBTR and DMFS. They obtained different cut-offs for tumours with different HR statuses (HR-positive: 49 months, versus HR-negative: 33 months)[14].

Estimation of the prognostic effect of a time-dependent covariate with a standard Cox model is known to be potentially unsatisfactory [20], with the cut-off or the exact shape of the relationship between DMFS and time to IBTR being difficult to determine without specific models. The limitations of all these methods include the *a priori* definition of the shape of the relationship. A time-dependent covariate must be specified (Ln (t), 1/t, spline, polynome etc.) and a cut-off value provides a restrictive shape for the relationship. The random forest approach is non-parametric and does not require the explicit specification of the shape of the covariate response. This makes it possible to extract the optimal relationship between DMFS and time to IBTR.

This is the first study, to our knowledge, to have used a statistical non-parametric analysis method for the evaluation of prognostic factors in patients with breast cancer treated by NAC. Several studies have confirmed of the promise of RSF relative to Cox PH models for real datasets [22,26,27], and have reported a better performance for RSF than for Cox PH models on the basis of the prediction error criterion [34]. This approach can be used to identify risk factors for poor breast cancer survival. RSF deals automatically and coherently with the limitations of traditional Cox models, such as the assumption of proportionality [35], and does not require advance knowledge of the relationship (i.e. linear or nonlinear) of a variable to time [22].

Using this approach, we identified a time to recurrence of 50 months as the better threshold for differentiating between good and poor prognosis groups. Early local recurrence probably reflects greater biological aggressiveness and/or higher resistance to treatment. This was particularly true for TNBC, which is widely recognised as having a good prognosis provided that no early recurrence occurs in the first five years after diagnosis. It is already known that recurrence patterns differ between molecular subtypes, with earlier recurrences for TNBC than for luminal tumours [36–38], but this study provides additional evidence that the late recurrence of TNBC is associated with a good prognosis. The main limitation of this study was the use of a method often considered a “black box” method, without comprehensive values (such as the hazard ratios of the Cox PH model) for interpretation. We used graphical methods to assess the predicted dependence of the response on covariates, and such interpretations may be subjective. However, graphical presentations facilitate comprehension of the interrelationship between covariates. Another point is the indication of radiotherapy. The French guidelines are, similar to many other national, regional and local guidelines, more often advising for post mastectomy radiotherapy (PMRT) compared to other more restrictive guidelines. In fact, we advice PMRT for all patients with involved axillary lymph nodes and to a selection of high-risk node-negative breast cancer patients. Our patient cohort concerns patients who had an indication for primary systemic therapy. Thereby, they had higher risk factors at diagnosis and thereby an indication for PMRT. Our study is homogenous in that all patients received primary systemic therapy and PMRT. We can thereby not compare with other patients groups but that wasn’t the intent of this study.

Our results suggest that time to IBTR should be seen as a key element for determining the patient’s prognosis. Our findings require validation in further studies, to determine whether patients with late recurrence have a similar prognosis to those with primary tumours. If this proves to be the case, then such late recurrences should be considered equivalent to a new primary tumour in terms of prognosis, for decisions concerning systemic treatments.

Conversely, patients with an early local relapse should be considered to have aggressive and resistant disease with a high risk of distant metastasis. These patients should be invited to participate in clinical trials assessing new therapeutic strategies, as initial chemotherapy failed to eradicate the tumour cells.
